# Ankle-Joint

**Published:** 1855-01

**Authors:** 


ANKLE-JOINT.—In operations at the ankle-joint, arrange your incisions so that you can gain access to the tarsal bones, and, if the disease be more extensive than anticipated, that you can even remove the entire foot at the joint, if you wish. Make "a transverse incision across the sole of the foot, commencing about three-quarters of an inch in front of one malleolus, and ending at a similar point in front of the other malleolus; a second incision is then made in the median line, beginning over the tendo-Achillis on a level with the ankle-joint, and joining the former at right angles in the sole of the foot. The two lateral flaps thus marked out being next dissected upwards close to the bones, the calcaneum and astragalus are freely exposed. By division of their ligamentous and tendinous connexions, one or both of these bones may be easily removed;" and other parts may be easily reached by extending the median incision a little forward. If from the great extent of disease it is found necessary to remove the entire foot, it may be done by uniting the two extremities of the transverse incision by a curved incision across the dorsum of the foot.

*Compound Fracture.*—When ought amputation to be resorted to where gangrene supervenes? Military surgeons, as Larrey and Guthrie, say you may amputate when the gangrene is spreading. Most surgeons say, delay till the gangrene has ceased to spread, and the line of demarcation has been fairly established. Perhaps the best practice is a middle course, viz:—if within a certain period no disposition to an arrest of the gangrene appear, we ought to operate while the patient is strong enough to bear it. Should, however, the gangrene spread quickly, accompanied by great prostration of strength, delirium, and rapid pulse, the operation is inadmissible.—*Braithwaite's Retrospect.*

